# 2D-based electrode materials for supercapacitors – status, challenges, and prospects

**DOI:** 10.1039/d4ra05473c

**Published:** 2024-10-18

**Authors:** H. H. Hegazy, Junaid Khan, Noshaba Shakeel, Eman A. Alabdullkarem, Muhammad Imran Saleem, Hussein Alrobei, I. S. Yahia

**Affiliations:** a Central Labs, King Khalid University, AlQura'a P. O. Box 960 Abha Saudi Arabia; b Department of Physics, Faculty of Science, King Khalid University P.O. Box 9004 Abha Saudi Arabia; c Department of Physics Government Postgraduate Collage No. 1 Abbottabad Khyber Pakhtunkhwa Pakistan junaidkhan.nanotech@gmail.com; d Department Of Higher Education Achieves and Libraries, Government of Khyber Pakhtunkhwa Pakistan; e Department of Physics, Abbottabad University of Science and Technology Khyber Pakhtunkhwa Pakistan; f Chemistry Department, College of Science, King Saud University Riyadh 11451 Saudi Arabia; g Department of Chemical and Biological Engineering, Gachon University 1342 Seongnam-daero Seongnam 13120 Republic of Korea imran.saleem@gachon.edu.kr; h Department of Mechanical Engineering, College of Engineering, Prince Sattam Bin Abdul Aziz University Al- Kharj 11942 Saudi Arabia

## Abstract

The pursuit of efficient and sustainable energy storage solutions has fueled significant interest in the development of advanced materials for supercapacitors. Among these, two-dimensional (2D) materials undoubtingly have emerged as promising candidates due to their unique structural and electrochemical properties. To address the inherent challenges such as restacking, limited ion-accessibility, limited scalability, stability under operational conditions, and the intricate balance between surface area and conductivity that hinder the practical application of 2D materials, this article delves into innovative approaches and emerging strategies and prospects that aim to enhance their performance and durability. A systematic exploration of synthesis methods, structural characteristics, and electrochemical performance as supercapacitor electrodes of key 2D materials, including graphene, MXenes, transition metal dichalcogenides (TMDCs), black phosphorous and phosphorene and their composites has been discussed. The discussion will extend to recent breakthroughs and innovations, shedding light on how researchers are leveraging the unique properties of 2D materials to overcome existing challenges in supercapacitor technology. Beyond mere documentation, this review seeks to inspire future research directions, foster interdisciplinary collaborations, and contribute to the ongoing evolution of energy storage technologies towards a more sustainable and efficient future.

## Abstract 2

To address the inherent challenges such as restacking, limited ion-accessibility, scalability, stability, and the intricate balance between surface area and conductivity, this article delves into emerging strategies and prospects.

## Graphical abstract

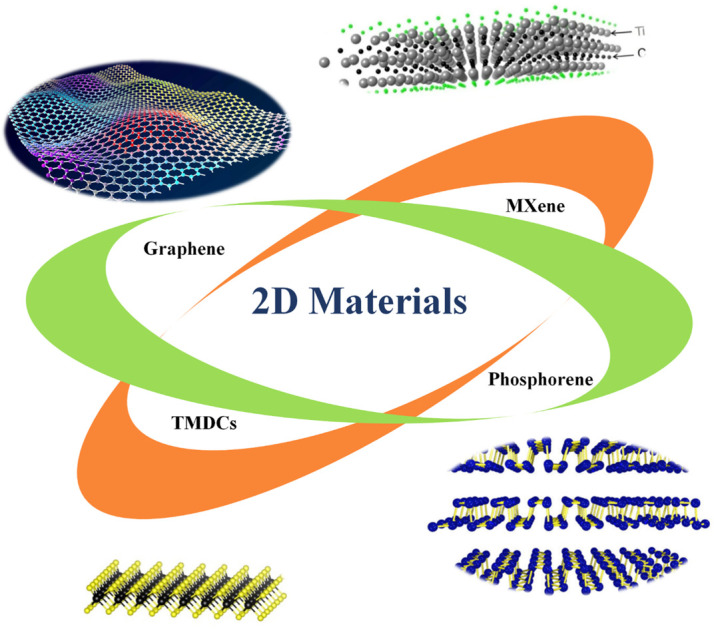

## 1. Introduction

The global energy consumption has exhibited an alarming upward trajectory, primarily propelled by the sustained combustion of fossil fuels.^1^ This trend has given rise to significant worldwide and environmental challenges, necessitating the pursuit of clean energy solutions for the establishment of a sustainable society.^2^ While renewable energy resources hold promise, their limited availability presents an impediment to achievability without the implementation of efficient and enduring energy storage systems.^3^ Supercapacitors (SC) and batteries represent prominent electrochemical energy-storage devices extensively utilized for corresponding tasks and are recognized as among the most auspicious storage systems. In the realm of batteries, the process of charge storage occurs *via* redox reactions occurring between active materials and the electrolyte. This confers upon them the capability for efficient specific energy (*E*_s_) enhancement. However, it also introduces vulnerabilities such as diminished cyclic performance and reduced specific power (*P*_s_).^4^ Electrostatic double-layer capacitors (EDLC) and pseudocapacitors are the two principal classifications of SC. EDLC involves the formation of an electrostatic double-layer through the adsorption/desorption of ionic species, facilitating charge storage.^5^ The electrostatic storage phenomena not only enable swift charge and discharge rates but also confer outstanding stability attributes to SC. Carbonaceous materials, including Graphene, Carbon nanotubes, and Activated carbon, serve as electrode materials for EDLC applications.^4^ Pseudocapacitance involves rapid and reversible redox reactions occurring at the electrode–electrolyte interface, thereby enabling supplementary charge storage beyond the double-layer capacitance [9]. Conducting polymers and metallic oxides exhibit pseudocapacitive behavior.^6^ Compared to batteries, supercapacitors store less energy per unit mass or volume, thereby constraining their viability as long-term energy storage solutions.^6,7^ The integration of hybrid chemistries, entailing a fusion of electrostatic and redox processes, within supercapacitor technology (hybrid supercapacitors) presents an avenue to attain synergistic capabilities between supercapacitors and rechargeable batteries.^8^ Recent developments in hybrid supercapacitors, distinguished by elevated *P*_s_, *E*_s_, and enduring cyclic stability, position them as a promising candidate for forthcoming energy storage technologies.^9^

**Fig. 1:**
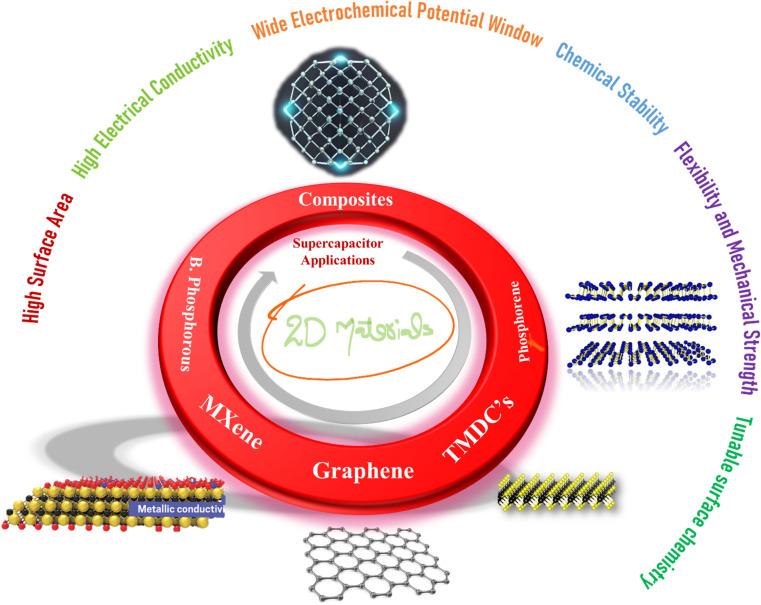
Schematic depiction of different 2D materials and their prospects for supercapacitor applications.

Carbon nanotubes, activated carbon, graphene, transition metal phosphatase, hydroxides, oxides, and metal–organic frameworks (MOFs) represent the most recently employed electrode materials for the aforementioned tasks.^10^ However, the straightforward implementation of these materials in practical assemblies has yet to yield the anticipated theoretical outcomes. Challenges such as limited storage capacity, slow reaction kinetics, cycling degradation, frail rate capability, and insufficient *E*_s_ have been pervasive, prompting the exploration of new categories of electrode materials.^11^ The quest for efficient, sustainable, and high-performance solutions has led researchers to explore innovative materials and design paradigms.^12^ Among the myriad of materials investigated for energy storage applications, two-dimensional (2D) materials have emerged as frontrunners, holding tremendous promise for revolutionizing supercapacitor technology.^13^ These materials, which include graphene, MXene, transition metal dichalcogenides (TMDs), black phosphorous and phosphorene, offer distinct advantages in the context of energy storage applications.^10,14–16^ Their inherently processed large surface area per unit mass, provides abundant active sites for ion interaction and their excellent conductivity ensures efficient electron transport within the electrode. Besides, they demonstrate remarkable mechanical strength, flexibility, advantageous for elastic and wearable supercapacitor applications.^17,18^ Additionally, their electronic properties can be tuned by adjusting the number of layers or through chemical modifications, which allow for the optimization of conductivity and capacitance according to specific requirements. Furthermore, their layered structure facilitates ion intercalation, enhancing ion accessibility to active sites and promoting efficient charge storage. These distinctive features have been schematically presented in Fig. 1.

Two-dimensional (2D) materials have captured significant attention for their potential to revolutionize the field of supercapacitors, thanks to their exceptional structural and electrochemical properties. However, despite their promise, several critical challenges persist in the practical application of 2D materials for energy storage. One of the foremost challenges is the scalability of these materials.^13^ Although methods like chemical vapor deposition (CVD) and exfoliation have shown promise in producing high-quality 2D materials, scaling up these techniques for mass production without compromising material integrity remains a substantial obstacle.^19^ The ability to fabricate large quantities of defect-free 2D materials is crucial for their integration into commercially viable energy storage devices. Moreover, stability under operational conditions presents another major concern.^20^ Many 2D materials, such as black phosphorus and certain transition metal dichalcogenides (TMDCs), exhibit instability in ambient environments, often undergoing oxidation, degradation, or phase transformations when exposed to air or during electrochemical cycling. Another intricate challenge lies in optimizing the balance between surface area and electrical conductivity.^21^ 2D materials like graphene offer high surface area, which is beneficial for charge storage, but they may lack sufficient electrical conductivity when assembled into bulk structures. On the other hand, some MXenes exhibit excellent conductivity but might not provide the surface area needed for high energy storage capacity.

This review aims to address these challenges by thoroughly examining the current progress and emerging solutions in the field of 2D-based electrode materials. We will explore how researchers are refining synthesis techniques to improve scalability and reduce defects, developing surface engineering strategies to enhance stability, and designing composite materials to achieve the desired balance. The conversation will also cover current developments and inventions, illuminating how scientists are using the special qualities of two-dimensional materials to get beyond current obstacles. This study attempts to give a thorough grasp of the present status of research on 2D-based electrode materials for supercapacitor applications by critically analyzing the literature and synthesizing the cumulative knowledge gathered in recent years. Beyond merely providing documentation, it aims to stimulate new lines of inquiry, encourage multidisciplinary cooperation, and support the continuous advancement of energy storage technologies towards a more efficient and sustainable future.

## 2. Graphene

A single layer with hexagonal arrangement of carbon atom, the graphene (G) possess a multitude of exceptional features, contributing to its whispered exploration for energy storage applications. Owning extraordinarily high surface area, that provide ample opportunities for adsorption of electrolyte ions at the interface makes them crucial contender for enhanced capacitance supercapacitors.^22^ The specific surface area of graphene typically ranges from 500 to 2630 m^2^ g^−1^, depending on its quality, number of layers, and the method of preparation. Single-layer graphene can have a specific surface area close to the upper limit, around 2630 m^2^ g^−1^. However, as the number of layers increases or if defects are introduced, the specific surface area decreases.^23–25^ The general synthesis approaches towards graphene are Chemical Vapor Deposition, Chemical Exfoliation, Electrochemical Exfoliation, Mechanical Exfoliation, and Thermal Reduction of Graphene Oxide. The two-dimensional structure of G ensures that a high proportion of its atoms are exposed at the surface, enhancing its electrochemical activity.^26^ Graphene and its derivatives primarily store charge through EDLC where charge storage occurs at the interface between the electrode and electrolyte without any faradaic (redox) reactions. The high surface area of graphene provides a large interface for charge accumulation, leading to high capacitance. Functionalized graphene (*e.g.*, with oxygen-containing groups) can also exhibit pseudocapacitance, where charge storage involves reversible faradaic reactions on the surface of the material. Besides, G exhibits exceptional electrical conductivity that allow efficient electron transport resulting in rapid charge/discharge process with low resistance and contributing superior power performance.^27^ G owns remarkable mechanical strength and flexibility which is most advantageous for maintaining the structural integrity of the electrode material during charge and discharge cycles, contributing to the long-term stability and durability. Its chemical inert nature avoid undesirable reactions with the electrolyte and ensure a stable electrochemical performance over time.^27^ Additionally, G owns high flexibility toward processability, allowing it to be easily integrated into various electrode architectures, including flexible and wearable devices. Because of these unique properties, G is positioned to become a material of choice for supercapacitors, helping to solve issues with energy storage, power delivery, and device miniaturization. G-based materials are still being optimized for ever more effective and adaptable supercapacitor technologies through ongoing research and developments.

**Fig. 2:**
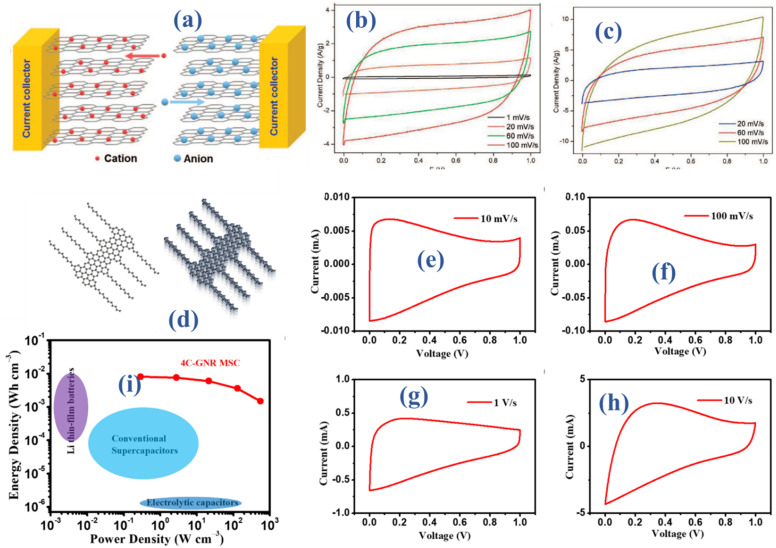
(a) Schematic illustration of fabricated device (b and c) CV elucidating EDLCs behavior^28^ (d) molecular arrangement of the utilized alkyl-substituted graphene nanoribbon (GNR) (e–h) CV at multiple scan rates (i) specific energy and power outcomes.^29^

The introduction of planar layers and atomically thin conductive materials, like Graphene (G), has indeed paved the way for revolutionary designs in thin-film energy storage devices, showcasing outstanding performance. A manufacturing method called “in-plane,” described by J. J. Yoo, entails producing ultrathin supercapacitors using pristine graphene electrodes in conjunction with multilayer reduced graphene oxide.^28^ Optimally utilizing the surface area of each graphene layer, implementing this in-plane configuration proves to be straightforward and enhances energy storage efficiency. In this study, two types of graphene oxide were used: pristine graphene, which was created by chemical vapor deposition, and multilayer graphene oxide, which was created by chemical reduction of graphene oxide films that were stacked layer by layer. For greater precision, pristine graphene and graphene oxide films were fabricated on quartz and copper foil substrates, respectively. The novel in-plane design creates a physical gap by cleaving graphene, in contrast to the traditional stacked architecture employed in supercapacitors, where graphene layers are arbitrarily oriented with regard to the current collectors. A huge conductive planar graphene sheet (2D) is efficiently divided into two electrodes by this approach. Subsequently, gold is sputtered on to the outer edges of the two electrodes to form current collectors. Fig. 2(a) illustrates the schematic device structure, while the electrochemical performance of the 2D configuration utilizing graphene was assessed through cyclic voltammetry (CV). Specific capacitance, which was obtained from the CV curves, was calculated between 1 and 100 mV s^−1^. The CV curves exhibit almost rectangular forms, as seen in Fig. 2(b) and (c), and they continue to do so at very high scan rates. Evidence suggests that an effective electric double layer (EDL) is formed in the graphene (G) electrodes. The thinnest devices, consisting of 1–2 produced graphene layers, may attain specific capacities of up to 80 μF cm^−2^, despite the open design and the impact of graphene edges. Multilayer reduced graphene oxide electrodes exhibit significantly higher specific capacities, reaching levels of up to 394 μF cm^−2^. Experimental investigations and model computations are employed to comprehensively explore the operational aspects of devices integrating both pristine and thicker G-based structures.

Graphene sheets have garnered attention due to their outstanding conductivity and substantial specific surface area, making them a promising option for flexible supercapacitors. Nevertheless, the utilization of these films is hindered by the trade-off between capacitance, which exhibits a positive correlated with structural defects, and electrical conductivity, which shows a negative correlation with structural defects. A composite film based on G was reported to be synthesized by the collaboration of G oxide and highly conductive G flakes.^30^ These elements work in concert to provide the composite films increased electrical conductivity, while graphene oxide adds even more pseudocapacitance to the mix. The final composite film has a remarkable value of 191.1 F g^−1^, which is higher than the specific capacitance of the pure graphene sheet (6.7 F g^−1^). Upon integration into a flexible supercapacitor, the composite film maintains consistent capacitance even under bending at different angles. Furthermore, even after undergoing 4500 cycles of bending and releasing (ranging from 0 to 180°), the supercapacitor retains a capacitance of 55.8%. A particularly interesting candidate for flexible supercapacitor applications is the composite sheet that integrates graphene with reduced graphene oxide, demonstrating remarkable electrochemical and mechanical performance.

Researchers have created few-layer graphene specifically for use in energy storage device applications by using a unique flame synthesis process on a metal current collector.^31^ A flame produced by a combination of pre-mixed gases, such as propane, oxygen, and argon, was used in a scalable, one-step technique to synthesize graphene. The utilization of sonication ensured the purity of the material and eliminated any undesired amorphous formations. This process successfully generated five to ten graphene layers on a nickel substrate. Requirements for rapid growth rates (between 30 and 60 seconds) included keeping a steady supply of precursors at a constant temperature. Activated carbon made from barley straw served as the active electrode material in the built supercapacitor, which also included a bought current collector. All electrodes maintained around 98% of their initial specific capacitance after 5000 charge–discharge cycles, as demonstrated by the results of the cycling stability test; the Ni/Flame-based graphene current collector, on the other hand, showed a significant decrease in capacitance. Thin graphene (G) may be added to a metal current collector in this study in an easy-to-scale manner to enhance the electrode's electrochemical properties. The success of this method is credited to the rapid growth rate and continuous processing capabilities of graphene (G). The study emphasizes the potential application of flame-synthesized few-layer graphene to enhance the electrochemical properties of supercapacitor electrodes. It also highlights large-scale production's feasibility and establishes it as a viable path forward for energy storage advancements in the future.

**Fig. 3:**
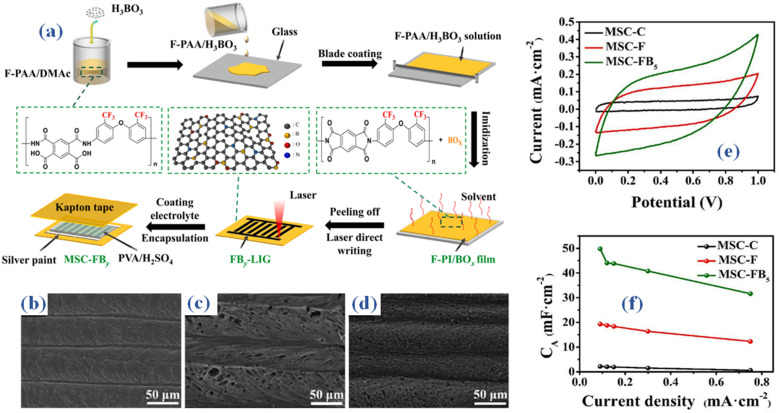
(a) Schematic representation of F-PI/Bo_*x*_ film fabrication process (b–d) SEM outcomes of fabricated films (e) the CV curves for MSC-C, MSC-F, and MSC-FB5 (f) arial capacitance at different current densities.^32^

The systematic fabrication of graphene nanoribbons (GNRs) through a detailed bottom-up synthesis, characterized by precise control over their width and edge structures, presents a compelling approach to overcome the inherent limitations of graphene. This approach demonstrates substantial potential in addressing a key limitation of graphene, specifically its inherent lack of an electronic bandgap. Simultaneously, graphene nanoribbons (GNRs) maintain the unique lattice structure of graphene in one dimension, while also exhibiting enlarged open-edge configurations in contrast to graphene. This attribute augments ion diffusivity, showcasing substantial promise for applications in energy storage systems. Nevertheless, G nanoribbons synthesized through current solution-based methods encounter noteworthy challenges related to substantial aggregation, primarily stemming from robust π–π interactions. This limitation hampers their broader applicability in practical scenarios. Hence, it becomes imperative to devise a straightforward and scalable methodology for the delamination of graphene nanoribbons (GNRs) from post-synthetic aggregates, resulting in the production of individual nanoribbons. A recent investigation introduces a high-shear mixing method aimed at dispersing graphene nanoribbon (GNR) bundles, effectively yielding individual GNRs through the incorporation of tailored molecular interactions.^29^ In Fig. 2(d), the molecular arrangement of the utilized alkyl-substituted graphene nanoribbon (GNR) is illustrated, featuring “cove”-type edges, alternatively referred to as gulf edges. Characterized by a width of four carbon atoms at their narrowest point, referred to as 4-CGNR, these edges boast lengths exceeding 200 nm. Normally, these 4-CGNRs present themselves in the form of aggregated bundle-like structures, as illustrated in Fig. 2(d). In this configuration, multiple 4-CGNRs densely pack together, giving rise to individual graphene nanoribbon (GNR) aggregates. The formation of these aggregates arises from robust π–π interactions, which exhibit resistance to prolonged sonication treatments. Given the effectiveness of high-shear mixing treatment in disrupting van der Waals interactions among 2D sheets to generate significant quantities of high-quality graphene, our proposition involves expanding this method to disperse the bundles of 4-CGNRs. Cyclic voltammetry (CV) measurements were carried out over a spectrum of multiple scan rates within a potential range of 0 to 1 V, in order to assess the electrochemical performance of the constructed micro-supercapacitors (MSCs) (see Fig. 3 (e–h)). Micro-supercapacitors with 4-CGNR microelectrodes often display a nearly rectangular CV curve, even under the harsh operating condition of 10 V s^−1^. This observation underscores the outstanding capacitive performance, primarily attributed to electric double-layer capacitance. This trait stems from the retained graphene structure, characterized by exceptional electronic properties. The apparent distortion observed in the cyclic voltammetry (CV) curves in Fig. 3(e–h) primarily originates from the resistance of the micro-supercapacitor (MSC). This resistance is attributed to the somewhat constrained electrical conductivity of 4-CGNR, in contrast to graphene materials obtained through top-down methods. Remarkably, these micro-supercapacitors (MSCs) exhibit unmatched operational speeds, reaching rates of up to 100 mV s^−1^. Ten times faster than the average sandwich-structured supercapacitor is this speed. This feature underscores their exceptional ultrafast charging/discharging capability. Specifically, microelectrodes using 4-CGNR have a volumetric capacitance (*C*_vol_) of 355 F cm^−3^ and a peak areal capacitance of 3.6 mF cm^−2^ at a scan rate of 10 mV s^−1^. The electrode used in micro-supercapacitors (MSCs) is made from a graphene nanoribbon (GNR) sheet that has been solution-processed. The impressive *P*_s_ of 550 W cm^−3^ is seen in Fig. 3(i). This level of performance matches, if not beyond, the state-of-the-art capabilities of graphene (G) and related carbon materials. Thus, the findings demonstrate the great potential of GNRs as electrode materials for future energy storage applications.

**Fig. 4:**
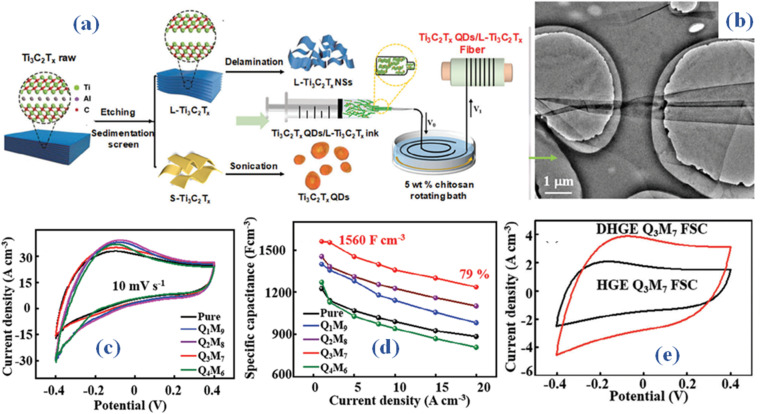
(a) Schematic representation of L-Ti_3_C_2_T_*x*_ fiber electrode fabrication process (b) SEM outcomes (c) CV curves 10 mV s^−1^ (d) the specific capacitance trend against different current densities, (e) CV of delaminated montmorillonite/polyvinyl alcohol (PVA) dimethyl sulfoxide (DMSO) flexible hydrogel (F-MMT/PVA DHGE) electrolyte devices.^33^

In scientific research, laser-induced graphene (LIG) has become a popular choice for micro-supercapacitor electrode materials. Still, a recurring challenge is the low capacitive performance of laser-induced graphene (LIG), which is ascribed to a restricted specific surface area and a lack of active sites. In this study, B. Cheng and associates cleverly used a laser direct writing method to *in situ* dope laser-induced graphene (LIG) with fluorine and boron atoms. The material used as a precursor for this process is fluorinated polyimide doped with boron, abbreviated as FB-PI.^32^ Scientists synthesized a boron-doped fluorinated polyimide (F-PI/BOx) layer in order to produce the precursor needed for fluorine and boron-doped laser-induced graphene (FB-LIG). An equal molar ratio reaction between 6FODA and PMDA in DMAc solvent for ten hours resulted in a solution that included twenty weight percent solid fluorinated polyamic acid (F-PAA). Then, using an adjustable film applicator (BEVS1806B), several weight ratios (1%, 3%, 5%, and 7%) of H_3_BO_3_ to F-PAA were added, well mixed, and placed onto a spotless glass slide. When the solvent was evaporated at 80 °C for two hours, a thermal curing operation was performed utilizing a predefined heating sequence (100 °C for one hour, 200 °C for one hour, and 250 °C for thirty minutes) at a heating rate of 10 °C min^−1^. A 60 μm-thick fluorinated polyimide/boron oxide (F-PI/BO_*x*_) film was produced as a result of this process. This was followed by the F-PI/BO_*x*_ film being subjected to the laser direct writing technique utilizing a commercially available blue and purple laser engraving equipment (Zovarpin, DK2500). This process required modifying the laser beam's power settings, engraving depth, scanning speed, and wavelengths between 400 and 450 nm, as seen in the digital images shown in Fig. 4(a). The surface morphologies of C-LIG, F-LIG, and FB5-LIG were investigated using scanning electron microscopy (SEM) images. When compared to C-LIG (Fig. 3(b)), it's apparent that both F-LIG (Fig. 3(c)) and FB5-LIG (Fig. 3(d)) exhibit a greater abundance of pores. The gaseous byproducts of the laser direct writing process, which are the -CF3 groups emitted from F-PI and FB5-PI, are what cause the increased porosity. As a result, this procedure induces a higher level of porosity within the produced structures. As a consequence, this process facilitates the development of supplementary pore structures. The CV curves for MSC-C, MSC-F, and MSC-FB5 are shown in Fig. 3(e). We obtained the curves at a scan rate of 10 mV s^−1^. Surprisingly, MSC-FB5 has the largest enclosed CV area relative to MSC-C and MSC-F, suggesting higher electrochemical energy storage capacity. The MSC-C, MSC-F, and MSC-FB5 cyclic voltammetry (CV) profiles are displayed in Fig. 3(e) at a scan rate of 10 mV s^−1^. The resulting material is called porous fluorine and boron co-doped laser-induced graphene (FB-LIG), and it has more active sites and improved wettability. The combined effect of boron and fluorine doping is responsible for this significant increase in capacitive performance. When the weight ratio of boron to fluorine is properly adjusted, micro-supercapacitors (MSCs) using poly(vinyl alcohol) (PVA)/H_2_SO_4_ as the gel electrolyte and FB-LIG as the electrode may reach an amazing areal capacitance of 49.81 mF cm^−2^ at a current density of 0.09 mA cm^−2^. This Fig. 3(f) surpasses the areal capacitance achieved by micro-supercapacitors (MSCs) produced using commercially accessible polyimide (PI)-based laser-induced graphene (LIG) by a factor of 23. Additionally, it has a three-fold increase in capacitance compared to MSCs made from LIG based on fluorinated polyimide (PI). Promising prospects for flexible wearable microelectronics, micro-supercapacitors (MSCs) made of FB-LIG also show exceptional mechanical stability and flexibility.

Supercapacitors can have their specific capacitance and *E*_s_ increased by combining graphene composites with additional materials such conducting polymers, metal oxides, or nanocarbons to create hybrid structures. A recent study presents a novel electrode material distinguished by a core–shell intercalation structure.^34^ The material is crafted through a direct and scalable method, making it suitable for commercial replication. Polyaniline makes up the electrode material's outer layer, which encases a nuclear framework made of nickel oxide and manganese dioxide. The described core–shell configuration is subsequently incorporated into the G oxide laminated structure, resulting in the formation of a PANI@(MnO_2_ + NiO)@GO composite. A strong adsorption capacity is demonstrated by the synergistic combination of polyaniline, metal oxides, and G oxide, which leads to the formation of a stable three-dimensional framework between the shell and the core. G oxide also adds to the composites' bilayer capacitance, which results in several synergistic effects occurring simultaneously and enhanced charge storage capacity. The composite material showcases remarkable characteristics as revealed by electrochemical testing. In an arrangement with three electrodes and a 0.5 A g^−1^ current density, it exhibits a 396 F g^−1^ specific capacitance. Calculations indicate that in a supercapacitor with a 1 M Na_2_SO_4_ electrolyte, the composite should have an *E*_s_ of 43.2 W h kg^−1^ and a *P*_s_ of 249.99 W kg^−1^.

Modifying the electrical characteristics of G by different doping techniques in order to improve its capacitance and conductivity. To achieve certain electrochemical properties, doping with nitrogen, sulphur, and other heteroatoms has been investigated. Improving the electrochemical performance of carbon materials by nitrogen doping is one very successful method. In a recent work, a nitrogen-doped reduced graphene oxide (NrGO) electrode for supercapacitor applications was created by doping the surface layers of a graphene oxide (GO) film with nitrogen ions and then annealing the film in a nitrogen environment.^35^ As a result of this approach, the NrGO electrode's surface layers had evenly spaced nanopores with a 9.9% nitrogen concentration. The combined impact of these surface alterations greatly enhances the electrode's electrochemical activity. In instance, when evaluated at a current density of 0.5 A g^−1^ in symmetrical cells employing a 6 M KOH electrolyte, the specific capacitance increases from 148 F g^−1^ (in the original reduced graphene oxide (rGO)) to 270 F g^−1^ (in nitrogen-doped reduced graphene oxide (NrGO)). At a *P*_s_ of 191.7 W L^−1^, the volumetric capacitance of NrGO is 416 F cm^−3^, and its volumetric *E*_s_ is 14.3 W h L^−1^. This work presents a novel nitrogen ion implantation technique for producing G-based electrodes with enhanced electrochemical performance.

By creating more conductive networks and increasing specific surface area, three-dimensional G designs, including G aerogels or foams, may be designed to promote quicker ion transport and better charge storage. Carbon aerogel, recognized for its outstanding conductive porous carbon properties, faces limitations in mechanical strength and fatigue resistance, restricting its utility in flexible electronic devices. Creating a unique layered structure, a novel cellulose nanofiber/reduced graphene oxide carbon aerogel (BCRCA) was fabricated by subjecting it to thermal annealing subsequent to a bidirectional freezing process.^36^ The ultra-low density of 3.25 mg cm^−3^ and high specific surface area of 1595.3 m^2^ g^−1^ of BCRCA are the outcomes of its layered structure. Additionally, it demonstrates exceptional compression characteristics, retaining 91.3% of its stress over 100 cycles at 50% compression. These findings underscore its effectiveness as a material for supercapacitor electrodes.

Indeed, the current focus among researchers revolves around the development of flexible and stretchable graphene-based supercapacitors for seamless integration into wearable electronics and flexible energy storage devices. This involves designing G electrodes and electrolytes that maintain performance under mechanical stress.^37^ Investigating cutting-edge electrolytes, such as gel-based and ionic liquid electrolytes, to enhance the overall *E*_s_, and stability are anticipated.^38^ Advancing roll-to-roll manufacturing and scalable chemical vapor deposition (CVD) techniques, among other economically viable approaches, to produce G-based supercapacitors on a wide scale are considered with keen interest.^39^ Examining how G-based supercapacitors may be included into hybrid energy storage systems to enhance overall performance by combining their quick charge/discharge times with batteries high energy densities can be explored. Efforts are underway to optimize the performance, scalability, and adaptability of graphene-based supercapacitors to cater to a diverse range of energy storage and electrical device applications.^40^

In the context of supercapacitors, miniaturization is crucial for integrating these energy storage devices into compact, flexiable portable electronics, such as smartphones, medical implants, and other devices. The challenge lies in designing supercapacitors that are not only small but also retain high energy and power densities. Advances in materials science, particularly with nanomaterials like graphene, have enabled the development of micro-supercapacitors yet facing challenges. While graphene offers excellent electrical conductivity and a high surface area, achieving consistent, large-scale production of high-quality graphene remains difficult. Variations in the number of layers, defects, and the presence of impurities can affect the performance of miniaturized devices. In flexible and wearable applications, graphene electrodes must withstand repeated bending, stretching, and twisting without significant degradation. Combining graphene with other materials (*e.g.*, electrolytes, substrates) in miniaturized and flexible devices can be complex. The compatibility of graphene with these materials, in terms of both chemical stability and physical adhesion, is crucial for reliable device performance. Increasing the energy density of graphene-based supercapacitors often requires thicker or multilayered structures, which can compromise flexibility. Balancing energy storage capacity with the mechanical flexibility needed for wearable applications is a key challenge. Addressing these challenges will be crucial to unlocking the full potential of graphene in next-generation energy storage devices.

## 3. MXene

Possessing the conventional formula M_*n*+1_·X_*n*_T_*x*_, MXene (MX) is a kind of two-dimensional (2D) transition metal carbides, nitrides, or carbonitrides. The letters M stand for a transition metal, X for nitrogen or carbon, and T for surface terminal groups. Because of their two-dimensional layered structure and remarkable electrical conductivity, they have a huge specific surface area and enable efficient and quick electron transport. Ions may readily access the surface of MXs due to its layered nature.^41^ This enhances the overall performance of supercapacitors by facilitating the electrochemical processes involved in energy storage. The general synthesis approaches involve Selective Etching, Chemical Vapor Deposition, and Top-down Synthesis from MAX Phases. MXenes store charge mainly through pseudocapacitive behavior. Their surface terminations (*e.g.*, –OH, –F, –O) facilitate fast redox reactions, contributing to high capacitance and excellent rate performance. Additionally, the MXenes also exhibit EDLC behavior due to their conductive nature and large surface area. Moreover, MXs' strong mechanical properties and high degree of flexibility make them ideal for wearable or flexible supercapacitor applications.^42^ MX based supercapacitors have the promise to withstand a long time owing to the strong chemical stability and durability. Maintaining performance across several charge/discharge cycles necessitates its stability. By changing their surface terminations or adding new components to the structure, MXs' characteristics may also be customised. Because of its tunability, researchers can better tailor MX-based supercapacitors for particular uses, increasing their overall efficiency.^43^ Additionally, eco-friendly synthesis techniques are frequently used, and the materials may be biocompatible. MX initially struggled because to its narrow voltage window (less than 1.23 V) under moist circumstances, even though it possessed a very high volumetric capacitance.^44^ Research examined the use of MX materials in the more voltage-range-extensive ionic liquid electrolytes. The process of creating a Ti_3_C_2_T_*x*_ (MX) ionogel film by vacuum filtering is described in the paper. The ionogel sheet is intended for use as electrodes in supercapacitors that use 1-ethyl-3-methylimidazolium bis(trifluoromethylsulfonyl)imide, a pure ionic liquid electrolyte.^45^ The disordered structure and steady spacing of the Ti_3_C_2_T_*x*_ hydrogel sheet are achieved by immersing it in an ionic liquid electrolyte during the vacuum drying process. TFSI and EMI^+^ ions can more easily access the Ti_3_C_2_T_*x*_ surface in this form. In an EMI-TFSI electrolyte environment that was clean, a capacitance of 70 F g^−1^ was achieved at a scan rate of 20 mV s^−1^, encompassing a wide voltage range of 3 V. The electrochemical signature demonstrates high power performance, showcasing capacitive response even at elevated scan rates (500 mV s^−1^). This study expands the possible uses of MX-based supercapacitors by presenting the idea of using MX materials with a variety of ionic liquid electrolytes.

Quantum dots (QDs) incorporated within an L-Ti_3_C_2_T_*x*_ (large size Ti_3_C_2_T_*x*_) fiber electrode (Q3M7) was reported to exhibit notable capacitance and exceptional flexibility, achieved through a wet spinning technique.^33^ Large-sized Ti_3_C_2_T_*x*_ nanosheets (NSs), designated as L-Ti_3_C_2_T_*x*_, are obtained through the natural sedimentation of raw Ti_3_AlC_2_, involving etching and mechanical delamination. Utilizing an ultrasound method, pillar-like Ti_3_C_2_T_*x*_ QDs are fabricated. The procedural methodology for crafting the fiber electrode with Ti_3_C_2_T_*x*_ quantum dots (QDs) embedded in L-Ti_3_C_2_T_*x*_ nanosheets (NSs) is elucidated in Fig. 4(a). Using a suction filter, the multi-layered material is cleaned with deionized water until the suspension achieves a pH of around 6. After 20 minutes of manual agitation, the multi-layered material delaminates into nanosheets (NSs). The delaminated L-Ti_3_C_2_T_*x*_ nanosheets exhibit a nanoplate shape, as shown in Fig. 4(b). The size distribution statistics indicate that the average size of L-Ti_3_C_2_T_*x*_ NSs is 7.6–9.5 μm and QDs is ≈ 2.3 nm. The effects of different content of Ti_3_C_2_T_*x*_ quantum dots (QDs) on the performance of the QxM10-x composite fiber electrodes may be evaluated by methodically analyzing their electrochemical characteristics in a 1 M H_2_SO_4_ electrolyte. One may assess the impact of varying content of Ti_3_C_2_T_*x*_ quantum dots (QDs) on the QxM10-x composite fiber electrodes' performance by carefully examining their electrochemical properties in a 1 M H_2_SO_4_ electrolyte. This finding implies that adding Ti_3_C_2_T_*x*_ quantum dots (QDs) improves the generated QxM10-x composite fiber electrodes' specific capacitance. It also retains a series of redox peaks, as shown in Fig. 4(c), indicating intercalation/deintercalation akin to that of H^+^ ions and the surface redox processes of Ti_3_C_2_T_*x*_. The Q3M7 fiber electrode, as shown in Fig. 4(d), retained 79% of its capacity at 20 A cm^−3^ and showed a strong mechanical strength of 130 MPa and specific capacitance of 1560 F cm^−3^. An all-solid-state fiber supercapacitor that can function across a broad temperature range uses these Q3M7 fibers as its electrodes. Delaminated montmorillonite/polyvinyl alcohol (PVA) dimethyl sulfoxide (DMSO) flexible hydrogel (F-MMT/PVA DHGE), as seen in Fig. 4(e), is utilized in this configuration as the electrolyte and separator. After 10 000 cycles, the supercapacitor retains 97% of its capacity and has a volume-specific capacitance of 413 F cm^−3^ at 0.5 A cm^−3^. Additionally, it operates at a *P*_s_ of 311 mW cm^−3^ and achieves an *E*_s_ of 36.7 mW h cm^−3^, exhibiting outstanding capacitance and flexibility throughout a broad range of temperature from −40 to 60 °C. The construction and assembly of all-solid-state fiber supercapacitors with a broad operating temperature range is made feasible by the current work. It achieves the perfect balance between capacitive performance and adaptability.

**Fig. 5:**
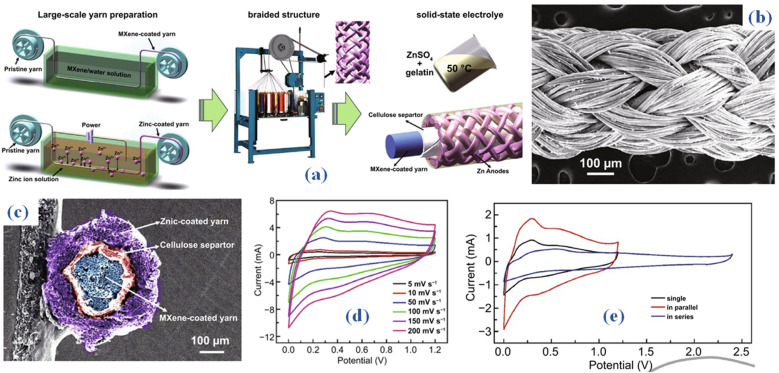
(a) Schematic representation of MX-based braided coaxial fiber fabrication process (b) SEM outcomes (c) The cross-sectional composition of the coaxial fiber supercapacitor (SEM) (d) CV curves encompassing scan speeds from 5 to 200 mV s^−1^ (e) CV of two coaxial FSCs arranged in series and parallel configurations.^47^

Zinc ion hybrid fiber supercapacitors (FSCs) are a potential type of energy storage devices for wearable electronics that stand out for their amazing qualities of high *E*_s_, flexibility, and wearability.^46^ Despite this, a notable challenge persists in optimizing the structure of FSCs to enhance *E*_s_ while simultaneously enabling the continuous fabrication of elongated FSCs. An innovative approach is introduced in a recent study, which proposes a braided coaxial zinc-ion hybrid fiber supercapacitor. This concept uses numerous meters of MX cathode as the core electrodes, then braids zinc fiber anode onto the surface of MX fibers over solid electrolytes to produce the shell.^47^ Through simulations conducted with ANSYS Maxwell software, it was observed that braided structures demonstrate superior capacitance in comparison to spring-like configurations. Fig. 5(a) depicts the step-by-step fiber fabrication process for constructing zinc (Zn) and MX-based braided coaxial fiber supercapacitors (FSCs). A specially designed semi-automatic apparatus was utilized to fabricate the Ti_3_C_2_T_*x*_ cathode through a dynamic multiple dipping process, involving the immersion of fibers into an MX suspension with a MX concentration of approximately 10 mg mL^−1^. The MX fiber cathode was able to obtain a mass loading of 0.15 mg cm^−2^ after completion of the drying procedure. Up to 1.5 meters was the maximum length that the MX fiber cathode could achieve. Using the corresponding in-house electrodeposition apparatus, the Zn fiber anode was produced *via* a continuous electrodeposition method. Next, a 35 μm-thick cellulose diaphragm was woven onto the surface of the fiber coated with Ti_3_C_2_T_*x*_ MX.

A network layer of tubular fabric was woven onto the cellulose diaphragm surface using zinc-coated fiber anodes and a 2D knitting machine to generate a basic texture. Three-layer coaxial fiber supercapacitor (FSC) outer layer: Six yarns were meticulously weaved in a clockwise fashion around the MX cathode, and six more yarns were woven in an anticlockwise direction, intersecting in pairs. Fig. 5(b), the SEM image of the manufactured coaxial FSC, makes the braided structure of the zinc anode clearly evident. The cross-sectional composition of the coaxial fiber supercapacitor (FSC) is depicted in the scanning electron microscope (SEM) image provided in Fig. 5(c). It is made up of a core MX cathode, a cellulose membrane, and a zinc (Zn) anode that resembles a shell. This arrangement gives the FSC a clearly defined, consistent, steady, and uninterrupted structure. After the electrochemical characteristics of the 2 cm long fiber supercapacitor (FSC) have been fully investigated, the cyclic voltammetry (CV) curves encompassing scan speeds from 5 to 200 mV s^−1^ are displayed in Fig. 5(d) within a voltage window of 1.2 V. The insertion of Zn ions into the Ti_3_C_2_T_*x*_ layers is responsible for the significant redox peaks seen in the lower potential range. This finding clarifies the dual energy storage process including double-layer effects and pseudoredox reactions. In particular, Zn is converted to Zn2+ during the Zn//Ti_3_C_2_T_*x*_ fibre device charging process. Zn then migrates from the anode to the cathode, intercalating into the Ti_3_C_2_T_*x*_ layers, or adhering to the MX cathode's surface. After 5000 cycles, the generated fiber supercapacitors (FSCs) maintain 83.58% of their capacity, demonstrating remarkable cycling stability. Furthermore, they demonstrate an astounding 214 mF cm^−2^ areal capacitance and 42.8 μW h cm^−2^*E*_s_ at a 5 mV s^−1^ scan rate. Two coaxial FSCs were carefully arranged in series and parallel configurations to increase the *E*_s_ and widen the potential windows, as shown in Fig. 5(e). When two coaxial fiber supercapacitors (FSCs) are connected in both series and parallel configurations, they display dual voltage windows and currents on cyclic voltammetry (CV) curves, while maintaining consistency across various scan speeds. This illustration demonstrates the capability to regulate output voltage and capacitance by utilizing multiple coaxial fiber supercapacitors (FSCs) arranged in both series and parallel configurations. Consequently, the designed fiber supercapacitors (FSCs) exhibit the ability to meet the energy and power requirements essential for practical applications in energy storage devices. Moreover, the coaxial fiber supercapacitor experiences different knot forms, demonstrating its potential to store fiber energy in a shape-controlled manner. In addition, outstanding wearability and stability are displayed by the knitted fiber supercapacitor (FSC). This makes it easier to include into watch straps or include it into fabrics to power LED arrays and smartwatches for several days at a time.

**Fig. 6:**
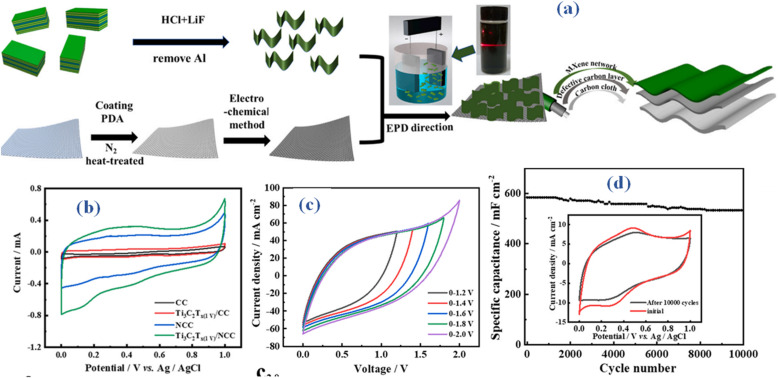
(a) Method illustration for producing nitrogen-doped superhydrophilic carbon cloth based binder-free Ti_3_C_2_T_*x*_ (EPD)/ENCC device (b) CV comparison (c) CV of Ti_3_C_2_T_*x*_ (EPD)/ENCC at different potential windows (d) cyclic life performance with inset revealing CV outcomes before and after 10 k cycles.^48^

In spite of the advancements achieved with Ti_3_C_2_T_*x*_ MX as a supercapacitor electrode material, there exists the potential for further enhancement of its capacitance through a composite approach. A new technique for producing nitrogen-doped superhydrophilic carbon cloth (ENCC), which is produced by doping carbon cloth (CC) with nitrogen, is presented in a recent study.^48^ Based on electrophoretic deposition, MX nanosheets were used as the supercapacitor electrode material to create the binder-free Ti_3_C_2_T_*x*_ (EPD)/ENCC, as illustrated in Fig. 6(a). The composite electrode yielded an area-specific capacitance of 2080.1 mF cm^−2^ at a current density of 1 mA cm^−2^. Based on the study, different Ti valence states are thought to be responsible for a certain pseudocapacitance within the electrode's capacitance. Despite this, the electric double layer capacitance (EDLC) continues to be the main contributor, as seen by the CV curves' rectangular shape (Fig. 6(b)). A broad voltage window of 1.8 V was also displayed by the built-symmetric supercapacitor, as seen in Fig. 6(c). At a current density of 20 mA cm^−2^, it also demonstrated good cycle stability, maintaining capacitance at 91%. To summarize, the effective dispersion of MX flakes was facilitated by employing a composite technique, leveraging the flexible substrate surface (Fig. 6(e)). The abundance of functional groups (^−^OH, –F, *etc.*) on the surface of MX makes it the perfect electrode material for electrochemical capacitors. By means of hydrogen bonding interactions, these groups interact with the carbon layer simultaneously. The wettability and electrochemical characteristics of MXs can be improved by functionalizing their surfaces with different groups (such as the hydroxyl, carboxyl, or amino), which would increase the performance of supercapacitors.

The impediments posed by the inherent self-stacking phenomenon induced by weak interactions and structural failure resulting from surface oxidation significantly impede the viable deployment of MXs in practical applications. A novel approach based on biothermochemistry to fabricate a three-dimensional (3D) crosslinked structure of Ti_3_C_2_T_*x*_ (referred to as 3D-MX) was recently reported.^49^ This innovative 3D-MX architecture is synthesized utilizing five distinct biological reagents, demonstrating commendable versatility. Post crosslinking, the 3D-MX material not only mitigates the challenge of layer stacking but also exhibits exceptional on-shelf stability lasting up to 550 days. It was elucidated that the mechanism underlying the formation of 3D-MX originates from electrostatic and chemical interactions between surface functional groups and the crosslinking agents. Remarkably, the Ti_3_C_2_T_*x*_ MXs subjected to biothermochemical treatment display an extraordinarily rapid film-processing capability, requiring only 3 minutes. This addresses the longstanding issue of inefficiency and laboriousness associated with the solution-vacuum filtration method for producing two-dimensional films. The 3D-MX architecture-used supercapacitors that were created showed a very high specific capacitance of 265 F g^−1^ at 1 A g^−1^. Furthermore, these supercapacitors demonstrate no discernible attenuation in capacitance over a period of 720 hours. The proposed biothermochemistry method for the facile and expeditious construction of a 3D Ti_3_C_2_T_*x*_ crosslinked network provides a novel perspective for advancing the practical application of MXs. This approach simultaneously addresses the challenges posed by self-stacking and oxidative degradation, offering potential avenues for enhancing the performance and viability of MXs in real-world applications.

In an attempt to create hybrid structures that take use of the complementing qualities of many materials, MXs have been integrated with other materials, such as metal oxides or conductive polymers. Enhancements in specific capacitance, stability, and conductivity can be obtained using these hybrid materials. A composite film consisting of MX and polypyrrole (M-PPy) was created through the vacuum-assisted suction filtration process. In this process, MX nanosheets were combined with polypyrrole (PPy) nanofibers.^50^ By intercalating PPy nanofibers, the arrangement of MX nanosheet layers is improved, reducing MX self-stacking and simultaneously lowering PPy swelling. Outstanding capacitance (563.8 F g^−1^ at 0.5 A g^−1^) and cycling performance (79.5% capacitance retention over 6000 cycles at 5 A g^−1^) are displayed by the optimized composite with 3 mL of PPy (M-PPy3). Making a flexible MnO2//M-PPy3 asymmetric supercapacitor (ASC) using MnO_2_ as the positive electrode and M-PPy3 as the negative electrode comes next. After 6000 charge/discharge cycles at 2 A g^−1^, the (ASC) exhibits an astounding 86.8% capacitance retention, providing an *E*_s_ of 35.3 W h kg^−1^ at 486.1 W kg^−1^. This highlights an outstanding capacity for energy storage. Moreover, the practical application potential of this system is demonstrated by successfully illuminating a 1.8 V LED through the connection of two ASCs in series. This investigation not only advances the understanding of the electrochemical performance of MX through an improved electrode structure but also highlights the applicability of M-PPy3 in flexible wearable devices, accentuating its versatility in emerging technological domains.

The electrochemical performance of MXs may be greatly influenced by adjusting their structural characteristics, such as the interlayer spacing. Doping MX structures with certain elements can change their electrical characteristics and result in better capacitive behaviour. To establish fine control over the electrode's structure and enhance overall device performance, researchers have been exploring novel approaches for the production of MX-based electrodes, such as 3D printing or inkjet printing.

## 4. TMDCS

Transition metal atoms are arranged in a hexagonal lattice within a plane between layers of chalcogen atoms, the transition metal dichalcogenides (TMDCs) exhibit several advanced features that make them promising candidates for supercapacitor applications. They provide a large specific surface area, especially in their two-dimensional (2D) shape. This characteristic increases the capacitance and energy storage capacity of supercapacitors by providing a high number of active sites for charge storage. By changing the number of layers or using particular TMDC materials, one may tailor the bandgap of TMDCs. Optimizing the charge storage capacity is made possible by this tunability.^51^ TMDs typically store charge through pseudocapacitance. The transition metal atoms in these materials can undergo redox reactions, facilitating high capacitance. Some TMDs also store charge *via* ion intercalation, where ions insert themselves between the layers of the material during charging and discharging. The general synthesis approaches involve CVD, liquid phase exfoliation, mechanical exfoliation and hydrothermal synthesis. The strong chemical stability and outstanding electrical conductivity of these materials, particularly in their monolayer form, are crucial for preserving the structural integrity of supercapacitor electrodes over repeated cycles of charge and discharge. TMDCs are well-suited for flexible and wearable supercapacitor applications because to their inherent mechanical strength and flexibility, particularly in their 2D form. The integration of TMDCs into supercapacitor devices and their scalable manufacture are made possible by the variety of synthesis procedures.^52^ The goal of ongoing research is to better comprehend and enhance TMDCs' electrochemical characteristics for use in energy storage systems.

Characterized by a mass loading of active materials that is economically substantial, supercapacitors are designed to offer higher energy storage capacities than their regular equivalents. The manufacture and performance assessments of a symmetric supercapacitor employing a commercially feasible 2D WS_2_ nanoflower electrode material with a mass loading of 30 mg cm^−2^ are presented for the first time by R. B. Rakhi. The investigation has impressive electrochemical properties 204 F g^−1^ of specific capacitance is attained at a scan rate of 5 mV s^−1^.^53^ The supercapacitor is particularly noteworthy for its remarkable *E*_s_ and power metrics, which measure 14 W h kg^−1^ and 0.8 kW kg^−1^, respectively, at 1 A g^−1^. The gadget has outstanding stability; it maintains 100% of its functionality for 10 000 consecutive cycles of charge and discharge. Supercapacitor coin cells that have electrodes with commercially relevant mass loading of 2D WS_2_ are also effectively used to light an LED, illustrating the usefulness of these cutting-edge energy storage technologies.

**Fig. 7:**
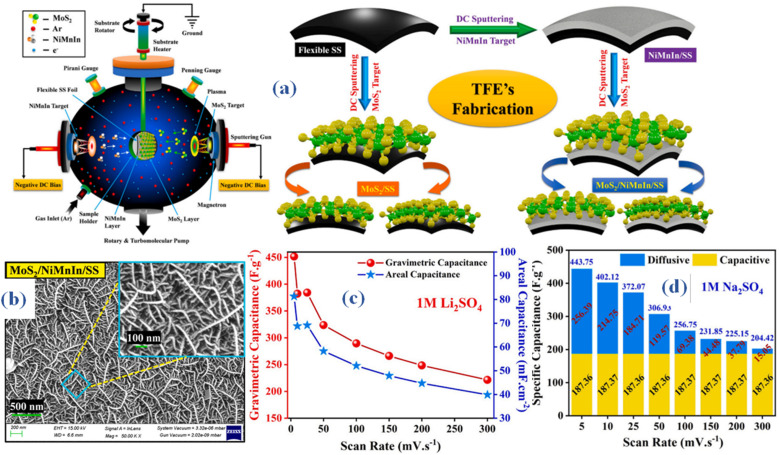
(a) Method illustration MoS_2_ nanowires in thin-film electrodes (TFEs) made *in situ* using the magnetron sputtering process (b) surface morphology outcomes (SEM) (c and d) specific capacitance using 1 M Li_2_SO_4_ and 1 M Na_2_SO_4_ as electrolyte respectively.^54^

The creation of a flexible supercapacitor device that incorporates NiMnIn shape memory alloy nanoparticles and MoS_2_ nanowires in thin-film electrodes (TFEs) made *in situ* using the magnetron sputtering process is described in a research report.^54^ MoS_2_/SS, NiMnIn/SS, and the MoS_2_/NiMnIn/SS heterostructure were the three electrode configurations that were created. Direct current (DC) magnetron sputtering was used to manufacture MoS_2_ and NiMnIn thin films, as shown in Fig. 7(a). Every thin layer was applied straight on the flexible, polished stainless steel (SS) current collector. The argon flow rate and the distance between the target and substrate were kept constant at 20 cm and 5 cm, respectively, throughout the sputter deposition procedure. Prior to deposition, a 10 minutes pre-sputtering process was applied to the shutter in order to remove any layers of surface contaminants from the target. Ultimately, the sputtering plasma was exposed to a 2 cm^2^ (2 cm × 1 cm) area of the SS substrate in order to promote the growth of MoS_2_ and NiMnIn thin films. Sputtering was directed towards the MoS_2_ in the MoS2/SS TFE, while in the NiMnIn/SS TFE, the NiMnIn was sputtered onto the bare SS substrate specifically. The MoS2/NiMnIn/SS TFE, on the other hand, was made by first sputtering the NiMnIn target onto the bare SS substrate for ten minutes, and then sputtering MoS_2_ over the NiMnIn bottom layer for fifteen minutes. At magnifications of 50 000× and 100 000×, the MoS_2_/NiMnIn/SS thin-film electrode (TFE) surface morphology was examined using scanning electron microscopy (SEM), as seen in Fig. 7(b). The study revealed homogeneous development of MoS_2_ nanowire-based 3D clusters that exhibited distinct open holes dispersed over the electrode surface. This finding points to the development of a very permeable and linked network. The thickness of the MoS_2_ nanowires ranged from around 10 to 20 nm, while their lengths varied from 100 nm for shorter nanowires to 1000 nm for longer ones. The distinctive 3D nanostructure contributes to an augmented specific surface area, creating numerous electroactive sites that enhance both ionic adsorption and redox kinetics. Electrochemical assessments demonstrated the superior pseudocapacitive charge-storage capabilities of MoS_2_–NiMnIn, particularly in terms of Na+ and Li+ kinetics. The notable improvement in performance is mainly due to the cooperative heterojunction creation of NiMn in nanoparticles and MoS_2_ nanowires. These heterojunctions lead to greater electrical conductivity, better substrate adhesion, increased specific surface area, and heightened ionic conductivity. Consequently, the flexible MoS_2_/NiMnIn/SS TFE demonstrated an outstanding specific capacitance of about ∼487.86 F g^−1^ (87.82 mF cm^−2^, 355.53 F cm^−3^) in a 1 M Na_2_SO_4_ electrolyte at 0.7 A g^−1^ (Fig. 7(c)). The all-solid-state flexible symmetric supercapacitor (SSC) device, using a PVA-Na_2_SO_4_ gel electrolyte, attained an operating current density of 0.56 A g^−1^, resulting in a peak cell capacitance of about 208 F g^−1^ (37.57 mF cm^−2^, 192.07 F cm^−3^). An impressive electrochemical stability was shown by the sodium ion-charged solid-state capacitor (SSC), which retained about 97.5% of its capacity after 6000 cycles. It also showed a favorable balance between high energy densities (about 28.99 W h kg^−1^) and power densities (about 12.81 kW kg^−1^). The capacitance values for a range of scan speeds, from 5 mV s^−1^ to 300 mV s^−1^, are displayed in the bar graph shown in Fig. 7(d). These values, as shown in the total capacitance, outline different behaviors resulting from surface-limited and diffusion-controlled kinetics. Additionally, after 1000 galvanostatic charge–discharge (GCD) cycles and +90° bending, the MoS_2_–NiMnIn material with a 3D nanostructure showed exceptional mechanical stability. Specifically, it kept the TFE operating at around 93.68% of its initial capacity and the SSC device operating at about 88%. This demonstrates the strong structural stability under mechanical strain of the 3D nanostructured MoS_2_–NiMnIn material.

**Fig. 8:**
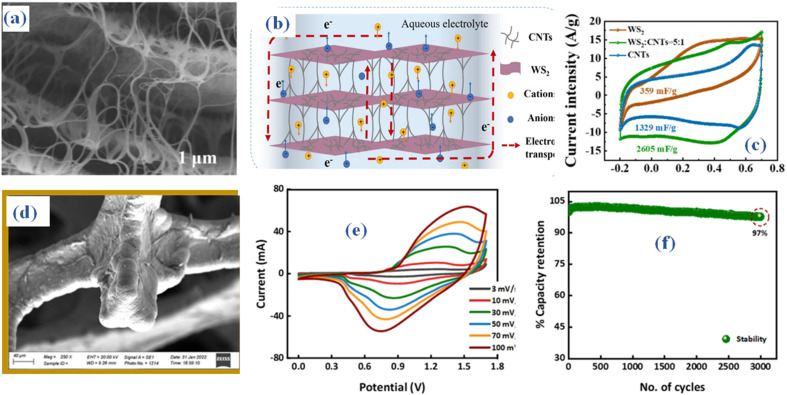
The WS_2_@CNT thin-film (a) SEM (b) schematic structure (c) CV comparison^55^ (d) magnetron sputtering MoWS_2_ SEM (e) CV (f) stability performance.^56^

It is imperative to develop a three-dimensional architectural framework for creating WS_2_ given the common limitations present in other two-dimensional layered materials, such as their vulnerability to stacking. This framework ought to be meticulously tailored to elevate its electrochemical performance. Furthermore, the constrained conductivity of WS_2_ poses limitations on its utility as a flexible electrode material. Using carbon nanotubes (CNTs) to enhance the hybrid WS_2_ materials' conductivity and provide a structural scaffolding for WS_2_ assembly is one way to overcome these challenges. The incorporation is evident in the scanning electron microscopy (SEM) images (Fig. 8a and b).^55^ The WS_2_@CNT thin-film hybrid, distinguished by its unique skeleton structure, achieves an exceptional specific area capacitance when compared to pure CNTs, according to a comparative study. This capacitance peaks at 752.53 mF cm^−2^ at a scan rate of 20 mV s^−1^, as shown in Fig. 8(c). Furthermore, the hybrid electrode material showcases exceptional stability, exhibiting a mere 1.28% decline in capacitance after 10 000 cycles. A quasi-solid-state flexible supercapacitor is created and put through extensive electrochemical testing to confirm its practical application. A specific area capacitance of 574.65 mF cm^−2^ is revealed by the data. Furthermore, the supercapacitor's elasticity is evaluated by bending it 10 000 times at an angle of 135°, which causes a 23.12% drop in capacitance at a scan rate of 100 mV s^−1^. These findings underscore its significant potential in this field.

Molybdenum tungsten disulfide (MoWS_2_) and WS_2_ are recognized as extremely promising electrode materials for energy storage devices due to their layered structures. In this context, the utilization of magnetron sputtering (MS) is imperative for depositing WS_2_ and MoWS_2_ onto the current collector surface, ensuring the achievement of an optimized layer thickness.^56^ An examination of the spewed material's morphological features was conducted using scanning electron microscopy (SEM) analysis (Fig. 8(d)). A hybrid device known as WS_2_//AC (activated carbon) was built after the synthesis of WS_2_ with the ideal thickness, which was shown to be the best-performing sample. The (CV) results of the hybrid device, depicted in Fig. 8(e), delineate capacitive behavior and discernible redox activities. The hybrid supercapacitor achieved a maximum *E*_s_ (*E*_s_) of 42.5 W h kg^−1^ and a *P*_s_ (*P*_s_) of 4250 W kg^−1^, demonstrating remarkable cyclic stability with a retention rate of 97% over 3000 consecutive cycles (Fig. 8(f)). In addition, *b*-values computed using Dunn's model indicate that the capacitive and diffusive properties seen throughout the charge–discharge process fall into the 0.5–1.0 range. This signifies the hybrid nature of the fabricated WS_2_ hybrid device. The remarkable performance metrics of WS_2_//AC make it highly suitable for prospective applications in energy storage.

## 5. Black phosphorous/phosphorane

A bulk allotrope of phosphorus that consists of layered sheets of phosphorus atoms, the black phosphorous (BP) exhibits unique properties that make it well-suited for energy storage devices, offering advantages over traditional materials. Its 2D nature results in a high surface area, allowing for more active sites for electrochemical reactions and contributing to enhanced capacitance in supercapacitors. It also possesses good electrical conductivity, with specifically tunable band gap.^57^ Additionally, anisotropic electrical and thermal conductivity, can be advantageous for designing electrodes with preferred charge transport pathways, leading to improved overall performance in supercapacitors.^58^ Liquid Phase Exfoliation, Mechanical Exfoliation and Plasma-Assisted Synthesis are the general synthesis approaches. To improve the overall efficiency of supercapacitors, scientists are investigating the integration of BP into hybrid frameworks with other components like metal oxides or carbon-based nanomaterials. In order to increase energy storage properties, these hybrid materials seek to maximize the advantages of each component.

**Fig. 9:**
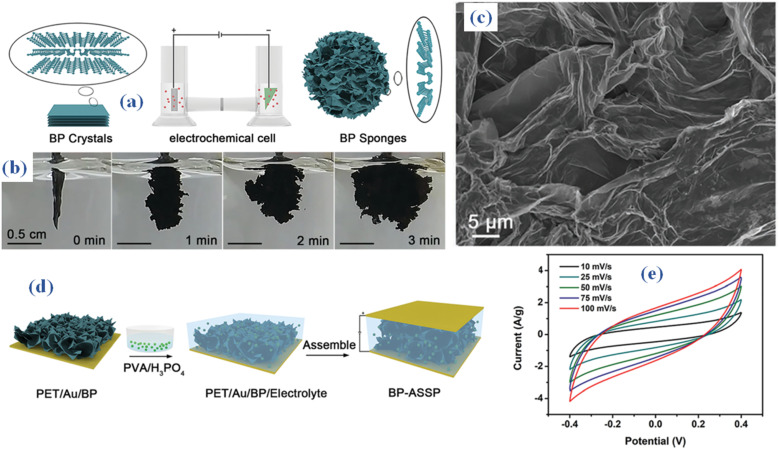
(a and b) Synthesis method of BP sponges from bulk BP crystals produces ultrathin nanosheets (c) SEM (d) electrolyte synthesis process, made of phosphoric acid and poly(vinyl alcohol) (PVA/H_3_PO_4_) (e) CV.^59^

Recently, it has been reported that electrochemical synthesis of BP sponges from bulk BP crystals produces ultrathin nanosheets with a thickness below 4.0 nm. These nanosheets exhibit substantial dimensions, surpassing tens of micrometers, and constitute the basic units of the sponge.^59^ An H-type electrolytic cell with an IT6123B direct-current power supply was used to carry out the electrochemical synthesis in Fig. 9a and b. Bulk BP crystals made up the cathode, while platinum plates constituted the anode. As the electrolyte, dimethylformamide (DMF) included 0.05 M tetrabutylphosphonium bromide. A separation between the cathode and anode was facilitated by a cation-exchange membrane. The produced BP sponges were removed and rinsed several times with DMF, acetone, *n*-hexane, and ethanol after the cell was run for three minutes at a voltage of five volts. The synthesized semi-connected nanosheets within the sponge create open channels that can be depicted from SEM (Fig. 9c). BP-ASSP, or an all-solid-state supercapacitor, is built by following the process shown in Fig. 9d. In summary, the BP sponge is broken up into tiny fragments and suspended in ethanol. The pieces are then applied to a PET substrate that has been coated with gold (Au), and the resulting BP sponge electrodes are then filled with a gel electrolyte made of phosphoric acid and poly(vinyl alcohol) (PVA/H_3_PO_4_). The CV results of the manufactured gadget are depicted in Fig. 9e. Using BP sponge as the electrode material, the all-solid-state supercapacitor outperforms both bulk BP crystals and BP nanosheets with a remarkable capacitance of 80 F g^−1^ at a scan rate of 10 mV s^−1^. As a result, BP sponges show great promise for energy storage applications.

Apart from that, the altered electrochemical method opens the door to improved exfoliation of other two-dimensional layered crystals, which in turn makes the creation of complex three-dimensional structures possible. The single or few layers of phosphorus atoms arranged in a puckered hexagonal lattice, similar to G, the phosphorene with its anisotropic electronic properties has gained attention for its potential applications in supercapacitors. Phosphorene stores charge predominantly through pseudocapacitance. The material's layered structure and high surface area allow for efficient ion intercalation and fast redox reactions, leading to significant energy storage capabilities. Exhibiting a high surface area due to its two-dimensional nature, and tunable bandgap make it advantageous for designing supercapacitors with tailored electrochemical properties. By adjusting the number of layers or applying external stimuli, such as strain or electric fields, the bandgap can be optimized for *E*_s_ storage requirements. The mechanical flexibility of phosphorene is another significant advantage for supercapacitor applications.^60^

Recently a single step using a chemical process was introduced by milestone in M. Gencten's study which was then integrated along polyaniline (composite).^61^ The production of composites involving polyaniline and phosphorene was documented for the first time, with these materials serving as electrode components in supercapacitors. To determine the ideal values for these characteristics in polymers, optimization of crucial electrochemical synthesis components was carried out, including cycle number, monomer concentration, and supporting electrolyte concentration. The resultant two-dimensional phosphorene was examined through the use of both microscopic and spectroscopic techniques to examine its morphological and structural characteristics. Subsequently, phosphorene was seamlessly incorporated into the structure of polyaniline (PANI) using an electrochemical method. The resultant materials, which were phosphorene-based, were used as supercapacitor electrode components. Hybrid supercapacitors (Phosphorene@PANI/PGEs) were characterized by means of cyclic voltammetry and electrochemical impedance spectroscopy methods. The study utilized the galvanostatic charge–discharge technique to investigate the impact of cycle number on the capacitive characteristics of these systems. The composite material doped with phosphorene exhibited a specific capacitance of 335.54 F g^−1^ when the charge–discharge current density was 1.6 A g^−1^. Following 1000 cycles under the same current density, the composite material exhibited a capacitance retention of 85%. In addition, after 4000 cycles at 2.5 A g^−1^ charge/discharge current densities, the retention rate was almost 80%. Remarkably, a significant rise in specific capacitance—259.21%—was noted in comparison to the electrode that did not include phosphorene. The system's specific capacitance was significantly increased by adding phosphorene to the conductive polymer framework.

Phosphorene has been synthesized using a variety of methods, such as chemical vapor deposition, liquid-phase exfoliation, and mechanical exfoliation.^62^ These techniques allow for the production of high-quality phosphorene with controlled thickness and properties, facilitating its integration into supercapacitor devices. Despite the promising characteristics, issues including scalability, affordable synthesis, and guaranteeing stability over time must be resolved. The development of scalable manufacturing techniques and the enhancement of the general performance of phosphorene-based supercapacitors are the main areas of ongoing research.

## 6. Synergy among composites

Researchers are looking at new materials and creative ways to improve the effectiveness of supercapacitors in an effort to advance energy storage technology. The composites' ability to combine several 2D constituents in a synergistic way offers an appealing means to customize supercapacitor electrodes with better properties. Scholars have been investigating the cooperative properties of composite structures made of several 2D materials in order to clarify the combined advantages that result from the interaction of these materials in the context of supercapacitor applications. Certain 2D materials, such phosphorene, transition metal dichalcogenides, and G, have unique benefits when it comes to energy storage due to their inherent features. Nevertheless, by utilizing the complimentary qualities of these materials, their use in composite constructions creates new opportunities.

A recent study, has employed a straightforward hydrothermal method with a two-step synthesis process to produce MoWS_2_ and its nanocomposite with Ti_3_C_2_T_*x*_ MX sheets.^63^ MoWS_2_ (MWS) nanosheets grew out of the MX sheets, which acted as templates and demonstrated impressive supercapacitive and electrocatalytic activity. At a particular current density of 0.2 A g^−1^, the MoWS_2_@MX (MWSM) had the largest specific capacitance ever measured, measuring 259 F g^−1^. Subsequently, in asymmetric studies, the MWSM was utilized as the negative electrode, while the CoSe_2_@CNT@rGO configuration was employed as the positive electrode. The resultant asymmetric device demonstrated remarkable cycling stability, sustaining coulombic efficiencies of 93% and 96% over 6000 cycles, with a *E*_s_ of 14 W h kg^−1^ and a *P*_s_ of 8 kW kg^−1^. The heightened electrochemical activity of the MoWS_2_@MX electrode was ascribed to its significant porosity, abundance of electrochemically active sites, rapid charge transfer kinetics, and unhindered pathways for ion transfer.

MX nanosheets are a very promising material for supercapacitor electrodes because of their exceptional redox activity and conductivity. However, obtaining high capacity and improved rate performance for MX nanosheets is difficult to balance, mostly because of their propensity to re-stack, particularly when there is a lot of mass loading. In this regard, Y. Yang has developed a 3D MX@G (MX@rGO) hydrogel that is characterized by increased ion accessibility, which is attained by MX and graphene oxide (GO) nanosheets self-assembling in response to aluminum.^64^ In order to fabricate hydrogels, the blunt aluminum oxide layer is etched using a diluted acid solution as the catalyst. This releases Al^3+^ ions, which serve as linkages between the nanosheets to form a three-dimensional structure. Concurrently, aluminum facilitates the reduction of graphene oxide, increasing the hydrogel's mechanical strength. A notable capacitance retention of 40.6% is demonstrated by the MX@rGO hydrogel, which has a peak area capacitance of 4.33 F cm^−2^ at 10 mA cm^−2^ and a sustained large area capacitance of 1.76 F cm^−2^ at 1000 mA cm^−2^. The hydrogel used to construct the asymmetric supercapacitor has remarkable cyclic stability, with just an 8.37% reduction seen after 100 000 cycles, highlighting its exceptional potential for real-world applications.

The layered structure of 2D materials often leads to restacking issues and poor electrical conductivity, limiting their overall performance. Past efforts have demonstrated the partial resolution of these challenges through composite electrodes comprising mixtures of G and MoS_2_, though their effectiveness is hampered by inadequate nanoscale mixing. The study introduces three composites synthesized using a straightforward ball-milling method and evaluates their performance as electrodes for supercapacitors.^65^ In comparison to pristine G and MoS_2_, the composites exhibit notably enhanced capacitance. In particular, the MoS_2_@G composite at a ratio of 1 : 9 exhibits a significant surface area and even MoS_2_ dispersion across the graphene sheet. MoS2@G (1 : 9) composite electrode outperforms the other two composites in an electrochemical supercapacitor, displaying an exceptional specific capacitance of 248 F g^−1^ at 5 A g^−1^. The created flexible symmetric supercapacitor device, on the other hand, exhibits remarkable flexibility and a long lifespan, holding onto 93% of its capacitance even after 8000 cycles. Remarkably, these scalable, freestanding hybrid electrodes exhibit consistent performance across varying angles, rendering them highly suitable for portable and wearable energy storage devices. They offer enhanced flexibility and superior supercapacitive performance, further emphasizing their utility in such applications. Table 1 elaborate the electrochemical performance of different discussed 2D material to provide an overview of current developments.

**Table 1:** Comparative analysis of 2D material to provide an overview of current developments

Material	Synthesis approach	Device type	S. capacity	S. energy	S. power	Stability (no of cycles)	Ref
Graphene flakes	Film	Flexible supercapacitors	191.1 F g^−1^			55.8% (4500)	30
Few-layer graphene	Flame-synthesized	Asymmetric supercapacitor				98% (5000)	31
Graphene nanoribbons	Bottom-up synthesis	Micro-supercapacitor	355 F cm^−3^		550 W cm^−3^	93% (10 000)	29
Boron and fluorine Co-doped graphene	Laser-induced	Micro-supercapacitors	49.81 mF cm^−2^	6.92 μW h cm^−2^	0.047 mW cm^−2^	93% (10 000)	32
Polyaniline/manganese nickel oxide/graphene	Simple scalable method	Three-electrode configuration	396 F g^−1^	43.2 W h kg^−1^	249.99 W kg^−1^	82.6% (3000)	34
Nitrogen doped reduced graphene oxide	Ions implantation	Three-electrode configuration	416 F cm^−3^	4.3 W h L^−1^	191.7 W L^−1^	Good stability	35
Cellulose nanofibers/reduced graphene oxide carbon aerogels	Bidirectional freezing technique	Supercapacitor	104.3 F g^−1^			93% (5000)	36
Ti_3_C_2_T_*x*_ quantum Dots/L- Ti_3_C_2_T_*x*_	Wet spinning method	All-solid-state	70 F g^−1^	36.7 mW h cm^−3^	311 mW cm^−3^	97% (10 000)	33
Zinc-ion MXene	Continuous fabrication	Fiber supercapacitors	214 mF cm^−2^	42.8 μW h cm^−2^		83.58% (5000)	47
MXene nanosheet N-carbon cloth	Electrophoretic deposition	Symmetric supercapacitor	2080.1 mF cm^−2^			91% (10 000)	48
MXene	Biothermochemistry method	Supercapacitor	265 F g^−1^			Good stability	49
MXene/polypyrrole	Vacuum-assisted suction filtration	All-solid asymmetric supercapacitors	563.8 F g^−1^	35.3 W h kg^−1^	486.1 W kg^−1^	86.8% (6000)	50
WS_2_	Hydrothermal technique	Symmetric supercapacitor	204 F g^−1^	14 W h kg^−1^	0.8 kW kg^−1^	100% (10 000)	53
MoS_2_ nanowires	Magnetron sputtering technique	Flexible supercapacitor	487.86 F g^−1^	28.99 W h kg^−1^	12.81 kW kg^−1^	97.5% (6000)	54
WS_2_@CNT	Thin-film	Solid-state flexible supercapacitors	752.53 mF cm^−2^	0.0798 mW h cm^−2^	5.745 mW cm^−2^		55
Black phosphorus	Electrochemical method	All-solid-state supercapacitors	80 F g^−1^				59
Phosphorene/polyaniline	Electrochemical synthesis	Hybrid supercapacitor	335.54 F g^−1^				61
MoWS_2_ and Ti_3_C_2_T_*x*_ MXene nanosheets	Hydrothermal method	Asymmetric supercapacitor	259 F g^−1^			98.72% (10 000)	63
MXene@graphene hydrogel	Al-induced self-assembly	Supercapacitor	4.33 F cm^−2^			80% (15 000)	64
MoS_2_/Graphene	Ball-milling method	Freestanding hybrid electrodes	248 F g^−1^			85% (1000)	65

### 6.1. 3D architectures using 2D materials

One of the key innovations in the field of supercapacitors is the development of three-dimensional (3D) architectures using 2D materials. These 3D structures aim to maximize the inherent advantages of 2D materials, such as high surface area, excellent conductivity, and tunable surface chemistry, while addressing issues related to stacking and limited active sites.

#### 6.1.1. Design strategies

Designing hierarchical porous structures with macro-, meso-, and micropores can facilitate ion transport, increase active surface area, and improve electrolyte accessibility. This can be achieved by combining different pore-forming techniques, such as template-assisted synthesis, chemical etching, and self-assembly methods. For example, using polystyrene spheres as a template can create macropores, while chemical etching can introduce mesopores and micropores.^66^

Creating interconnected networks of 2D materials can enhance electrical conductivity and mechanical stability. Techniques such as hydrothermal synthesis, solvothermal methods, and electrophoretic deposition can be used to assemble 2D materials into interconnected networks. For instance, graphene oxide can be reduced *in situ* to form a conductive network of graphene sheets.^19^

Developing 3D frameworks like aerogels, foams, and scaffolds from 2D materials can provide structural integrity and high surface area. Methods such as freeze-drying, 3D printing, and chemical vapor deposition (CVD) can create robust 3D frameworks. Graphene aerogels, for example, can be fabricated by freeze-drying a graphene oxide suspension followed by reduction.^67^

To foster innovation, future research should focus on combining multiple 2D materials (*e.g.*, graphene-MoS_2_ hybrids) to leverage the unique properties of each component in a 3D framework. Developing scalable and cost-effective synthesis methods for industrial-scale production of 3D architectures from 2D materials. Exploring functionalization techniques to introduce specific properties (*e.g.*, doping with heteroatoms) that enhance the performance of 3D architectures. Utilizing computational modeling to design optimal 3D architectures and predict their performance, guiding experimental efforts.

## 7. Significant advancement and potential future direction

Recent advancements in 2D materials for supercapacitor applications, as discussed in this review, have primarily focused on enhancing energy storage capacity, improving charge–discharge rates, and increasing material stability. Some notable breakthroughs are as follows: researchers have developed a variety of graphene-based composites aimed at improving supercapacitor performance. The integration of graphene with metal oxides or conducting polymers has resulted in significant enhancements in *E*_s_ and capacitance. The class of 2D transition metal carbides, nitrides, and carbonitrides, known as MXenes, has shown great promise due to their exceptional electrical conductivity and hydrophilic nature. Specifically, MXenes like Ti_3_C_2_T_*x*_ have been reported to create electrodes with high capacitance, and enhanced thermal stability and mechanical strength. Phosphorene, derived from black phosphorus, has garnered attention due to its high specific surface area and excellent electrical conductivity. Phosphorene-based supercapacitors have demonstrated remarkable energy storage capabilities. Molybdenum disulfide (MoS_2_) and other transition metal dichalcogenides (TMDs) are being investigated for supercapacitor applications owing to their unique electronic properties and large surface area. Hybrid materials that combine MoS_2_ with other conductive substrates have shown improved performance and good cycling stability. Creating heterostructures by stacking different 2D materials (such as graphene with MoS_2_ or MXenes) can synergize the properties of each component, leading to supercapacitors with superior performance metrics. Doping 2D materials with other elements (such as nitrogen or sulfur) or functionalizing their surfaces can significantly enhance their electrochemical performance and stability. These advancements in 2D materials are paving the way for next-generation supercapacitors with higher energy densities, faster charge–discharge cycles, and longer lifespans, making them more viable for a wide range of applications, from portable electronics to electric vehicles.

Our investigation into 2D materials for supercapacitor applications highlights their significant potential due to their unique properties such as high surface area, excellent electrical conductivity, and tunable chemical structures. However, to fully realize their potential, several challenges must be addressed, particularly in the areas of scalability, cost-effectiveness, and electrochemical efficiency. We propose that potential future research prioritize the following areas.

### 7.1. Scalability and cost-effectiveness

Future research should focus on developing novel, scalable, and cost-effective synthesis methods. For instance, chemical vapor deposition (CVD) and electrochemical exfoliation methods could be optimized for mass production while reducing costs. Utilizing abundant and sustainable raw materials can also significantly reduce production costs. Research into alternative precursors for the synthesis of 2D materials can pave the way for more economical production processes.

### 7.2. Electrochemical efficiency

Incorporating 2D materials with other nanostructured materials, such as metal oxides or conducting polymers, could enhance electrochemical performance by synergistically combining their properties. Functionalizing the surface of 2D materials with heteroatoms (such as nitrogen, sulfur, or phosphorus) can improve their electrochemical properties by increasing conductivity and active sites for redox reactions.

### 7.3. Device integration

Developing advanced electrode architectures, such as 3D porous structures or hybrid nanostructures, can enhance ion diffusion and electron transport, leading to better performance in practical supercapacitor devices. The integration of 2D materials into flexible and wearable electronic devices is a promising direction. Research should focus on optimizing the mechanical properties of 2D materials to maintain performance under deformation.

**Fig. 10:**
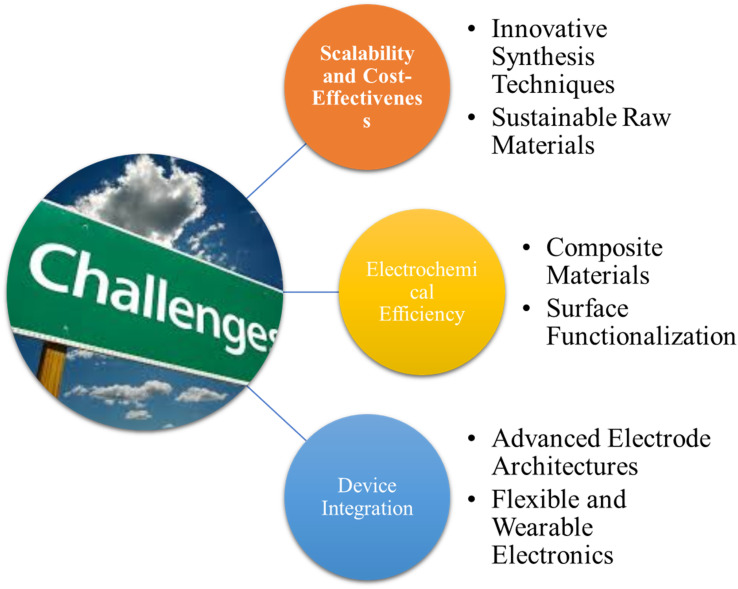
Challenges and future direction.

Fig. 10 schematically represents the paved future direction to the discussed challenges.

## 8. Conclusion

This comprehensive review aims to serve as a valuable resource for researchers, scientists, and engineers working in the field of energy storage, providing insights into the current state of knowledge, recent advancements, and the potential future directions of 2D-based supercapacitor electrode materials. The extensive exploration of graphene, MXene, transition metal dichalcogenides (TMDCs), and other emerging 2D materials has highlighted their unique properties, such as high surface area, excellent electrical conductivity, and mechanical flexibility, which make them promising candidates for advanced energy storage devices. The current state of research indicates significant progress in enhancing the performance of supercapacitors through the integration of 2D materials, showcasing improved *E*_s_, *P*_s_, and cycling stability. However, challenges such as scalability, cost-effectiveness, and electrochemical efficiency must be addressed to pave the way for practical implementation. Looking forward, the future direction of 2D materials continued research and innovation are expected to unravel novel synthesis methods, advanced hybrid materials, and scalable fabrication techniques, further propelling the development of high-performance supercapacitors.

## Data availability

The data will be made available on request.

## Conflicts of interest

The authors declare no financial conflict of interest.
